# Correction: Perceptual decisions are biased by the cost to act

**DOI:** 10.7554/eLife.26902

**Published:** 2017-03-27

**Authors:** Nobuhiro Hagura, Patrick Haggard, Jörn Diedrichsen

Hagura N, Haggard P, Diedrichsen J. 2017. Perceptual decisions are biased by the cost to act. *eLife* **6**:e18422. doi: 10.7554/eLife.18422.

Published 21, February 2017

We have detected a minor programming error for creating Figure 1E. For the error-bars on Figure 1E, instead of the S.D. divided by the square of the number of participants (S.E.) being presented, the S.D. was only divided by the number of participants without being squared. Figure 1E has been now corrected accordingly. No other aspects of our results, figures or interpretations are affected. We apologize for any confusion that may have occurred. We appreciate Ehsan Sedaghat-Nejad and his colleagues for pointing this out.

The corrected Figure 1 is shown here:

**Figure 1:**
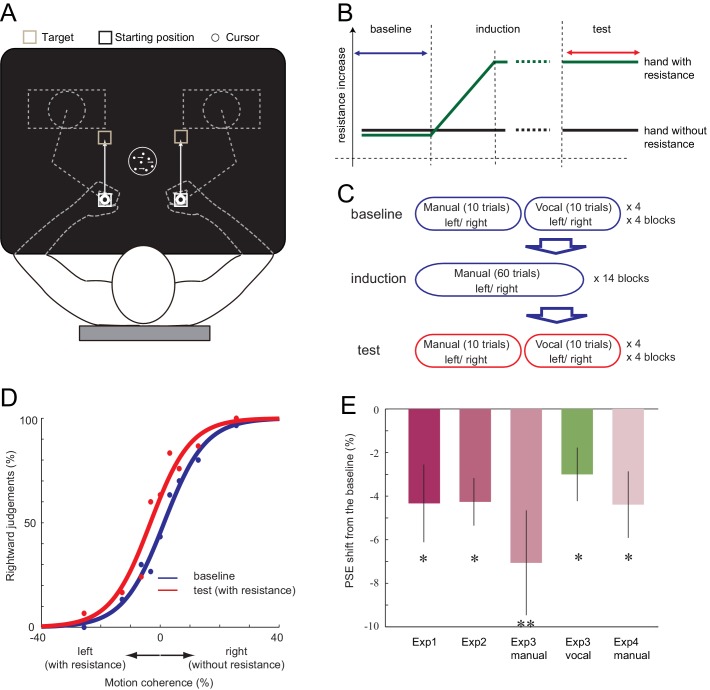

The originally published Figure 1 is also shown for reference:

**Figure 2:**
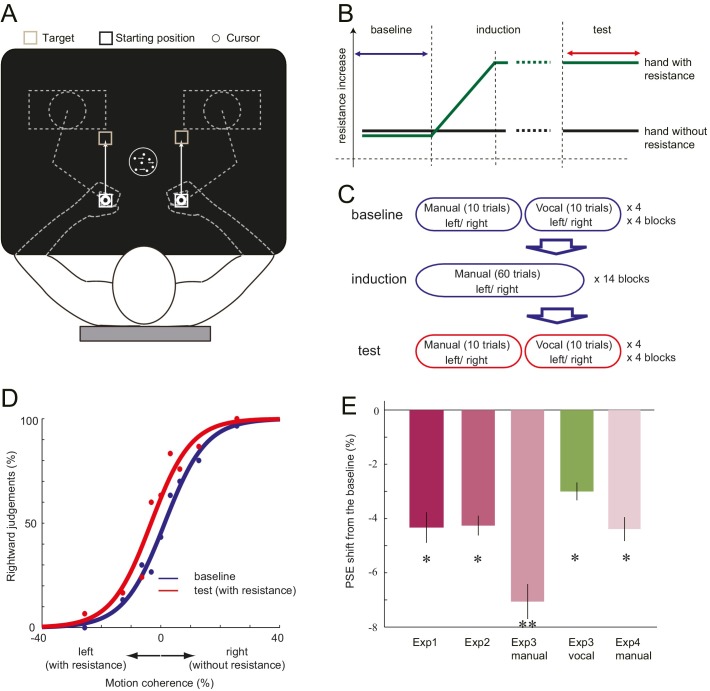

The article has been corrected accordingly.

